# APPLICATION OF A NEW TOOL FOR PLANNING IMPLEMENTATION EVALUATIONS IN CLINICAL TRIALS

**DOI:** 10.1093/geroni/igad104.2332

**Published:** 2023-12-21

**Authors:** Whitney Mills, Kali Thomas, Yuan Huang, Michael Wininger, Scott Hummel

**Affiliations:** VA Providence Healthcare System, Providence, Rhode Island, United States; Johns Hopkins University, Baltimore, Maryland, United States; VA CT Healthcare System, West Haven, Connecticut, United States; VA CT Healthcare System, West Haven, Connecticut, United States; VA Ann Arbor Healthcare System, Ann Arbor, Michigan, United States

## Abstract

The VA Cooperative Studies Program (CSP) study “Geriatric OUt-of-hospital Randomized MEal Trial in heart failure – Veterans Affairs” (GOURMET-VA) is a randomized, single-blind, multi-center, clinical trial investigating the effects of home-delivered meals in Veterans discharged from hospitalization for heart failure. GOURMET-VA is one of the first CSP studies to integrate implementation scientists during protocol planning. We describe how the three phases of the new Implementation Planning Assessment (IPA) Tool was used proactively to guide planning of the implementation evaluation for the trial, oriented towards future adoption and sustainment of the intervention. Phase 1 (“Planning, Framing, & Aligning Interested Parties”): During early protocol development, the implementation team helped identify and facilitated meetings with relevant VA national leadership. These meetings resulted in plans for engagement throughout the trial and input on trial design. Phase 2 (“Implementation Process Data Collection”): Guided by IPA, the implementation evaluation uses mixed methods to understand the context into which the intervention is being implemented, experiences of Veterans and clinicians with the intervention, and barriers and facilitators to implementation at the patient, healthcare provider, and leadership levels. Phase 3 (“Planning for Sustainment of Effective Trials”): A preliminary dissemination and sustainability plan was drafted, including how the implementation evaluation data may be used to inform adaptations of the GOURMET-VA intervention to improve effectiveness and uptake. We found that the structured guides that comprise the IPA were valuable in helping the implementation scientists and the trial team to find common ground in terms of rationale, methods, and language.

